# Supplementary figures and images for: Cellular characterization of the mouse collecting lymphatic vessels reveals that lymphatic muscle cells are the innate pacemaker cells
Source: eLife. 2025 Sep 11;12:RP90679. doi: 10.7554/eLife.90679 (PMC12425481; doi:10.7554/eLife.90679)

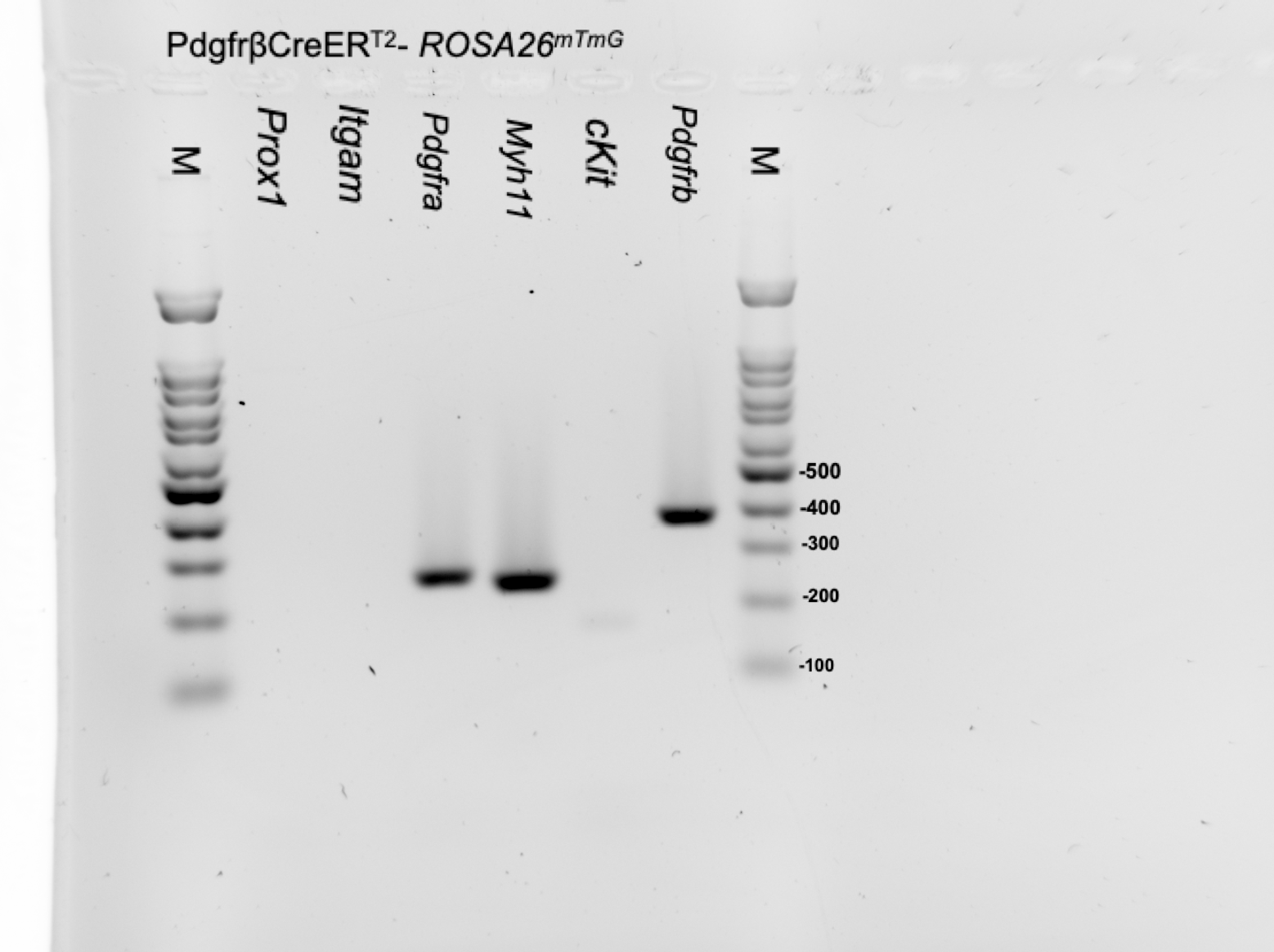

Supplement: Figure 4—source data 1. [file elife-90679-fig4-data1.zip › Figure 4 K-N Markup/Source File Figure 4N.tif]

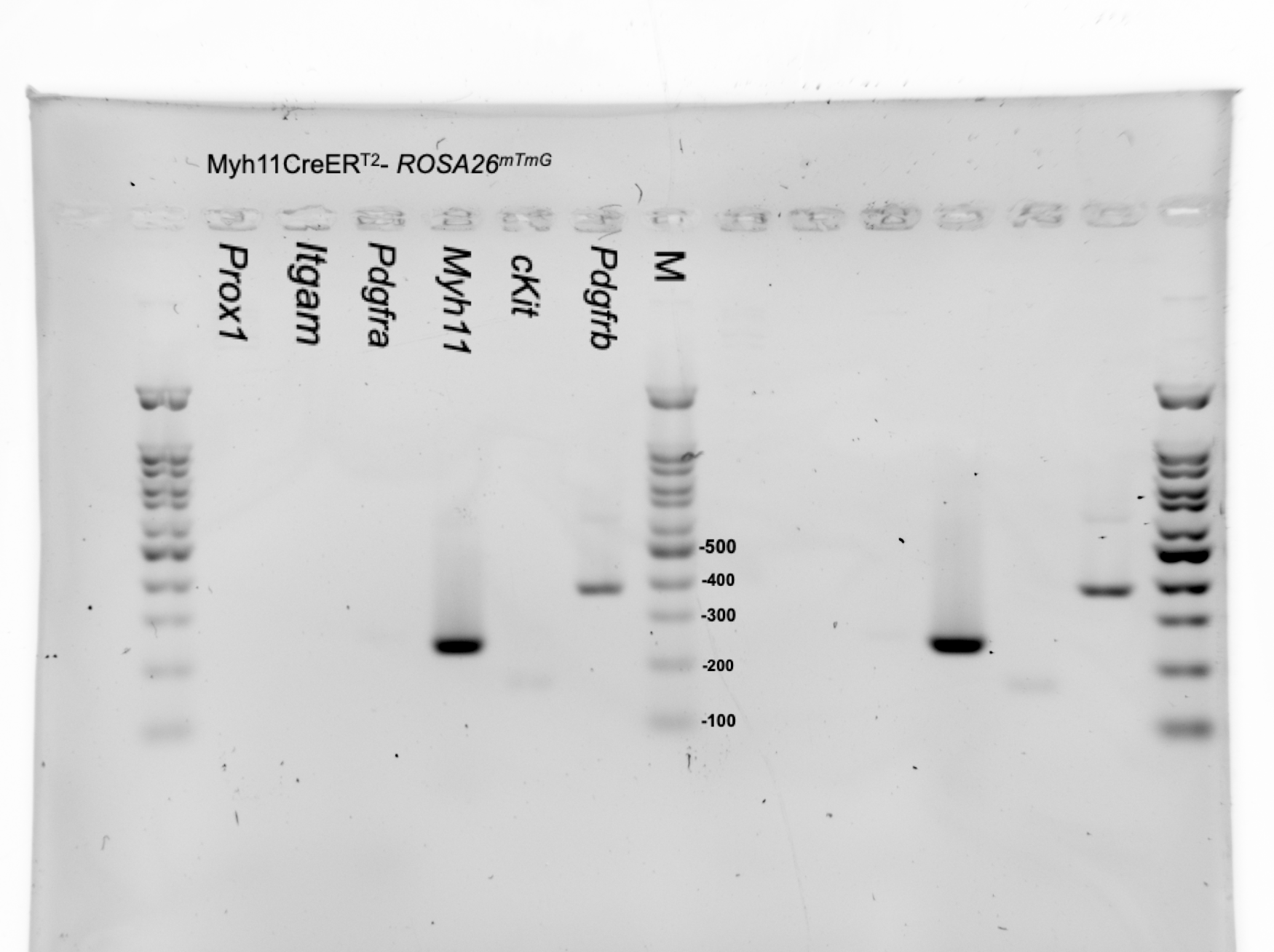

Supplement: Figure 4—source data 1. [file elife-90679-fig4-data1.zip › Figure 4 K-N Markup/Source File Figure 4M.tif]

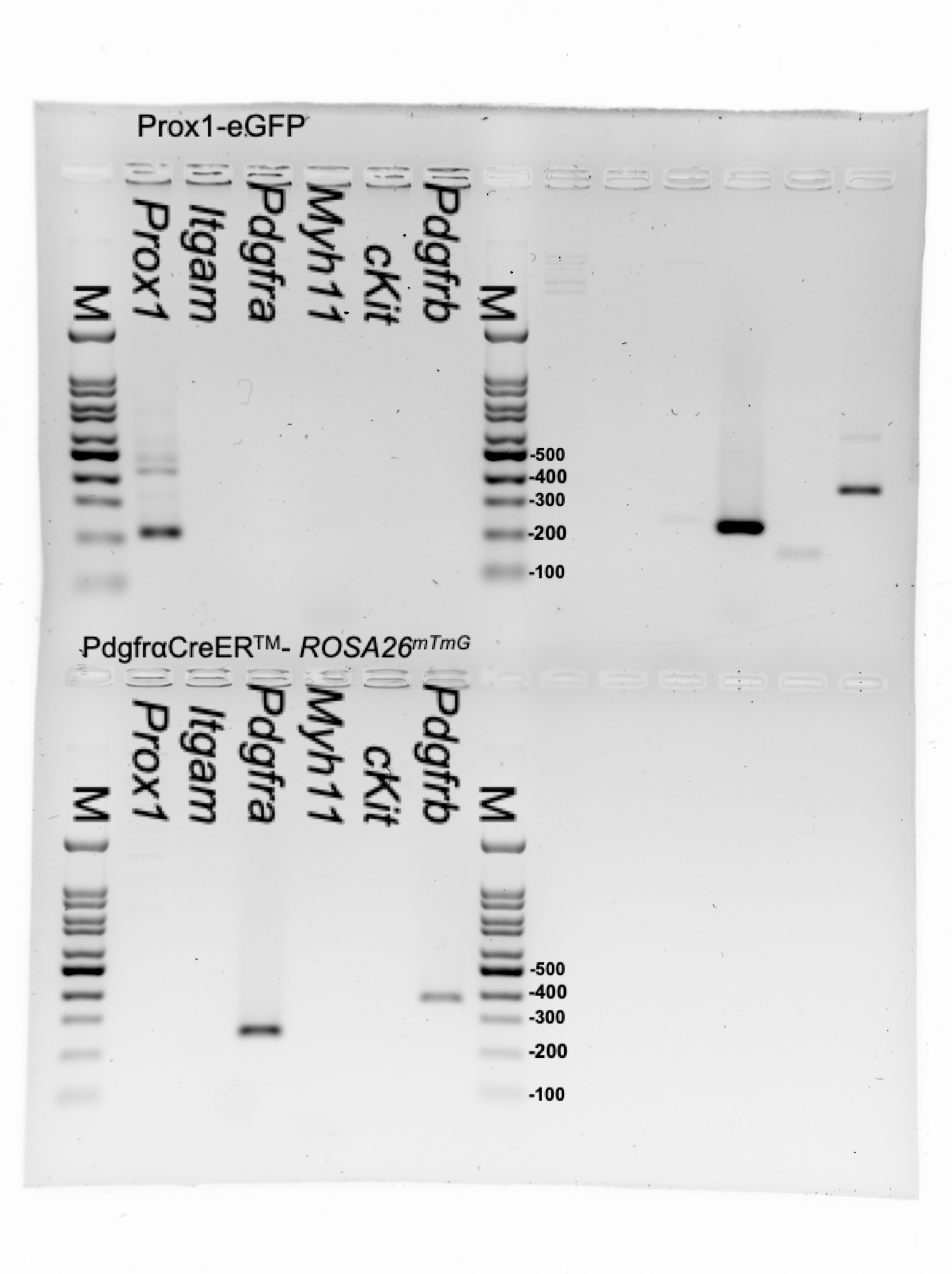

Supplement: Figure 4—source data 1. [file elife-90679-fig4-data1.zip › Figure 4 K-N Markup/Source File Figure 4K, L.tif]

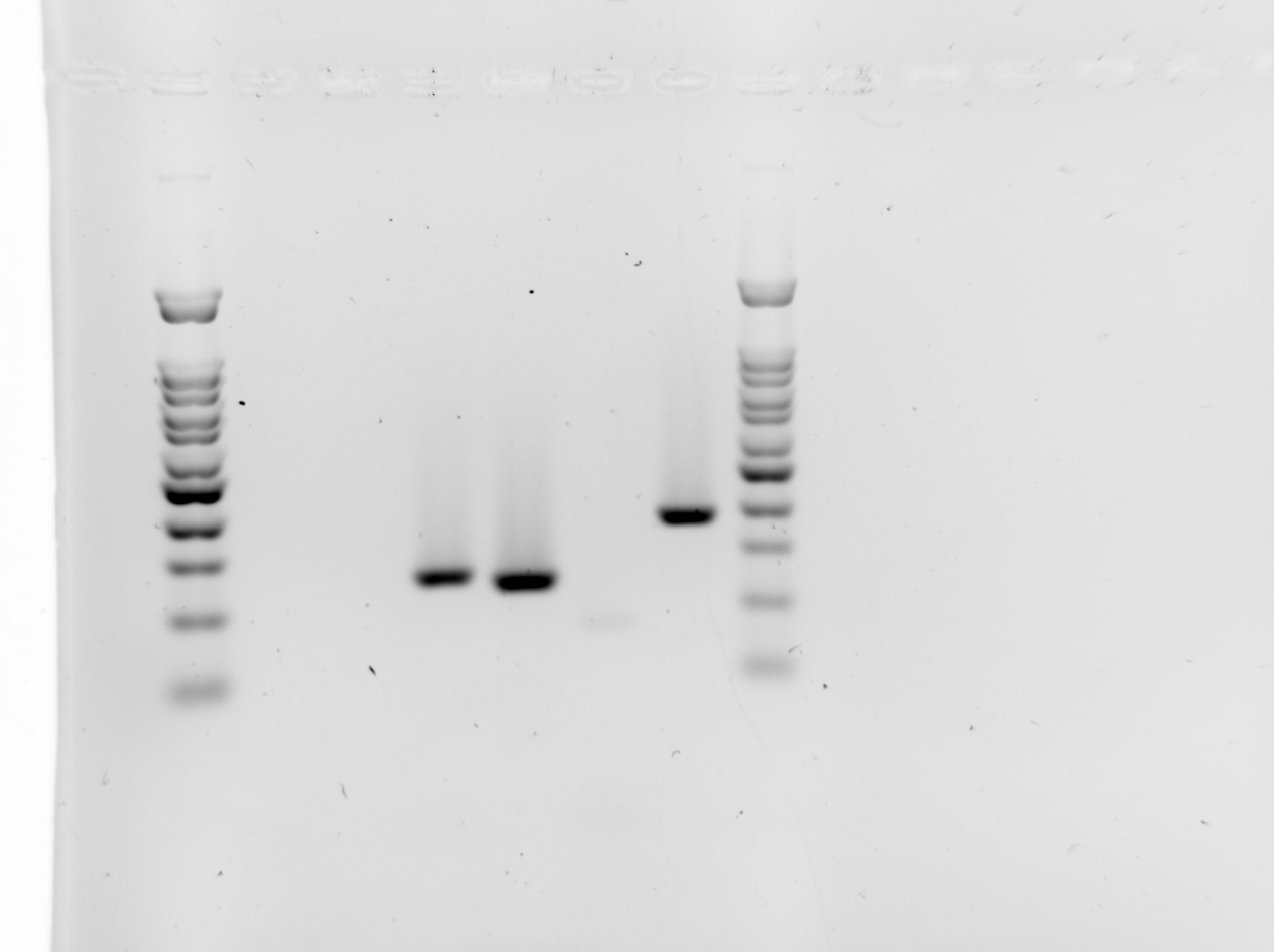

Supplement: Figure 4—source data 2. [file elife-90679-fig4-data2.zip › Figure 4 K-N No Markup/Source File Figure 4N.tif]

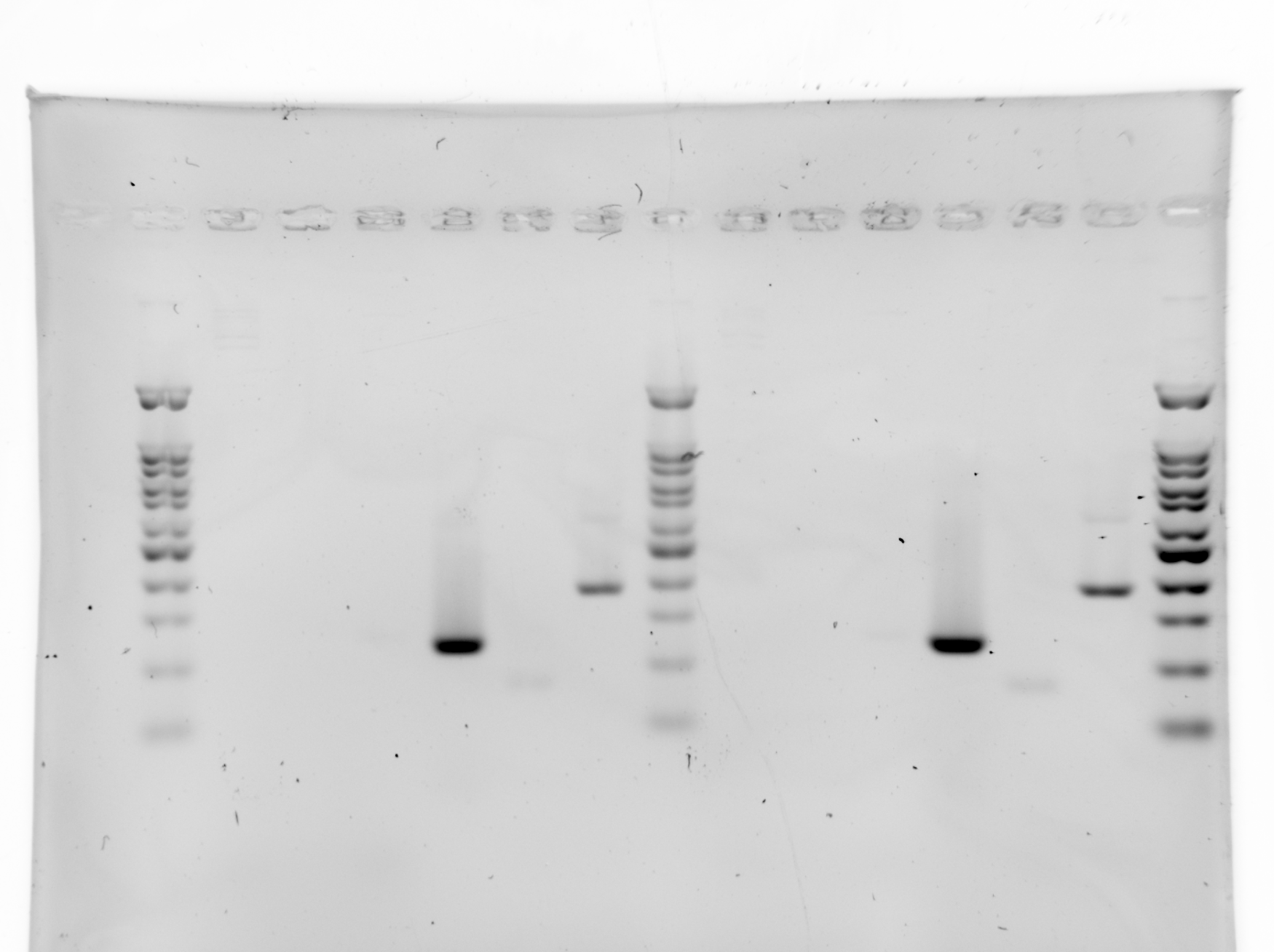

Supplement: Figure 4—source data 2. [file elife-90679-fig4-data2.zip › Figure 4 K-N No Markup/Source File Figure 4M.tif]

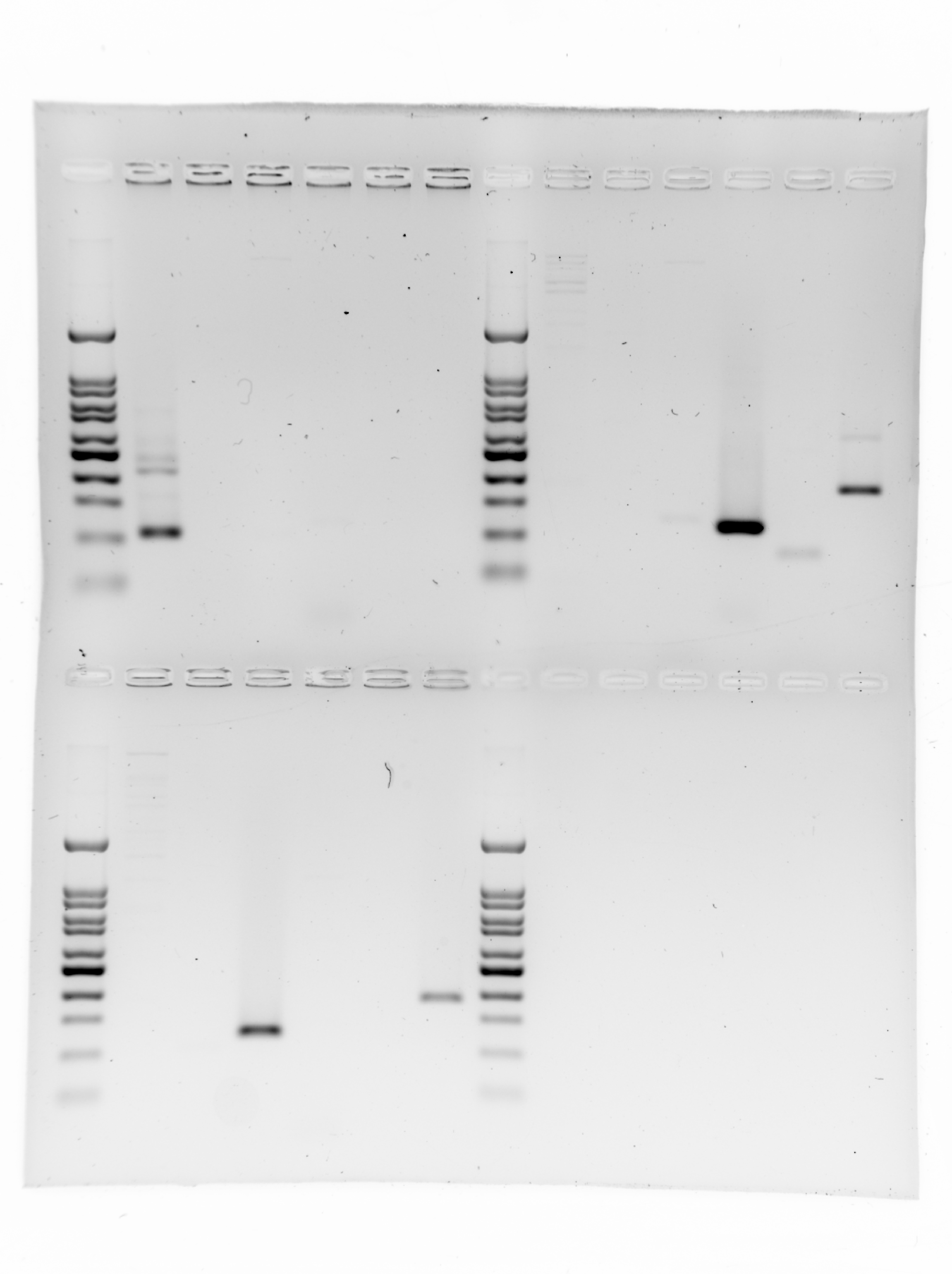

Supplement: Figure 4—source data 2. [file elife-90679-fig4-data2.zip › Figure 4 K-N No Markup/Source File Figure 4K, L.tif]

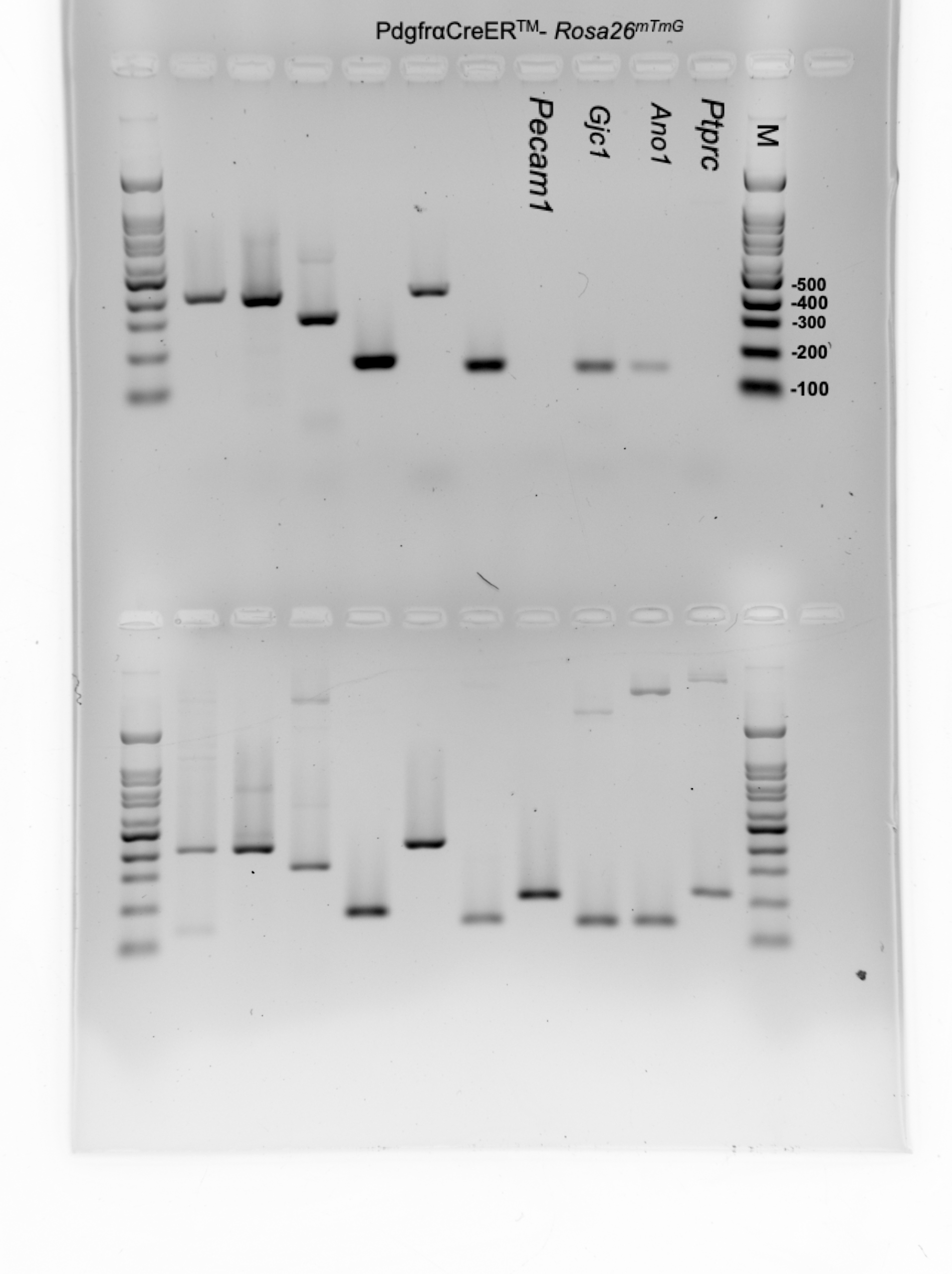

Supplement: Figure 6—source data 1. [file elife-90679-fig6-data1.zip › Figure 6 A-D, P Markup/Source File Figure 6P.tif]

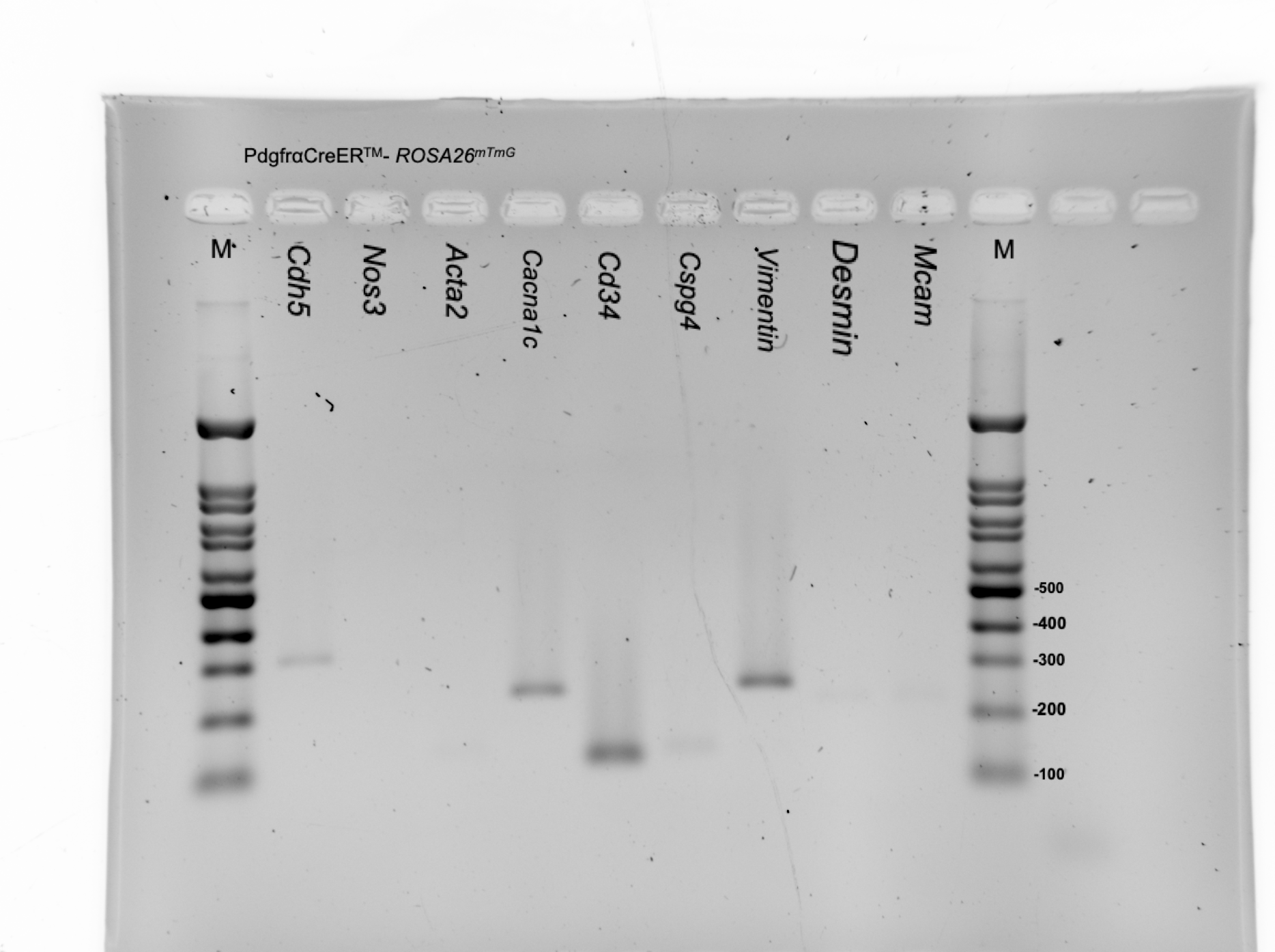

Supplement: Figure 6—source data 1. [file elife-90679-fig6-data1.zip › Figure 6 A-D, P Markup/Source File Figure 6D.tif]

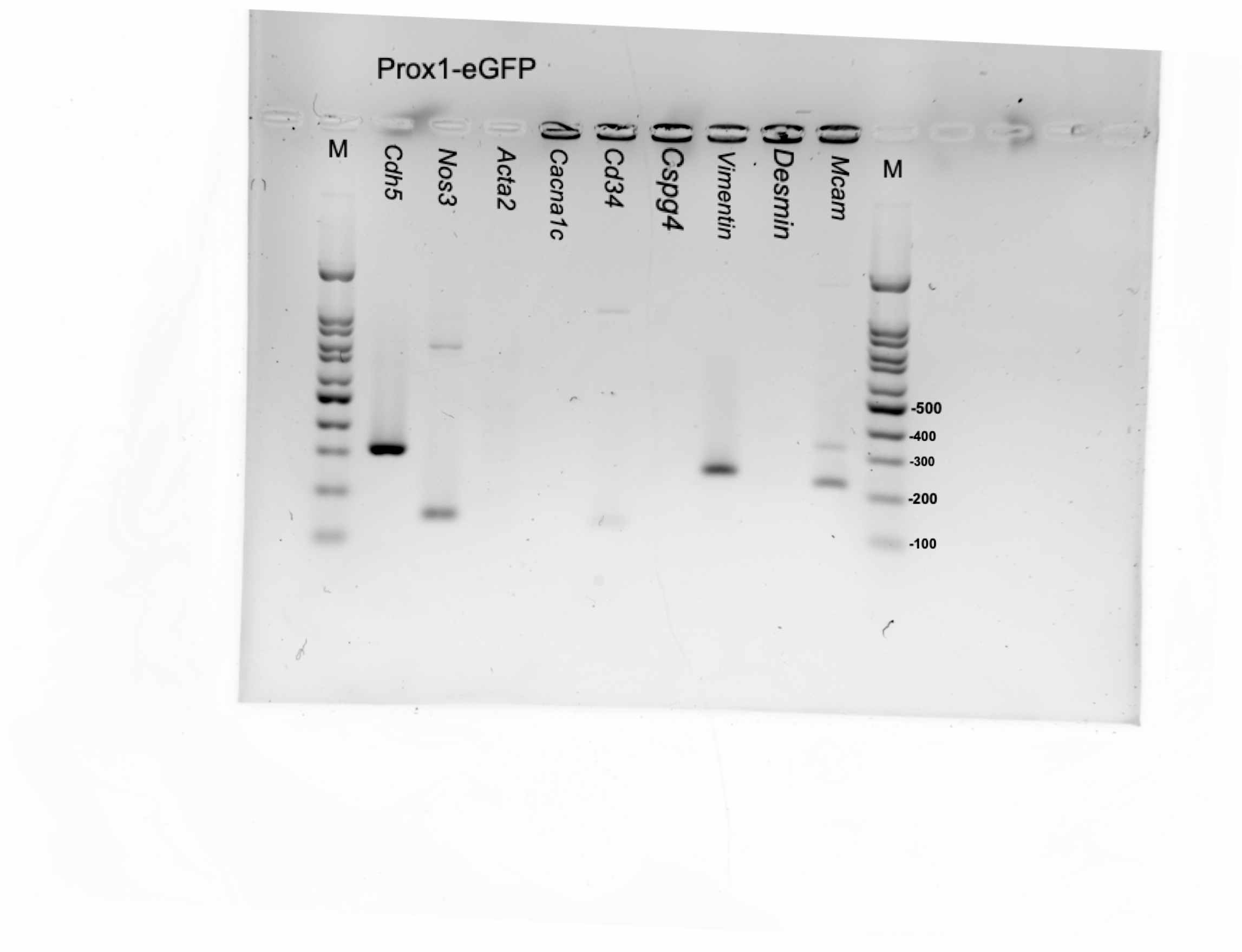

Supplement: Figure 6—source data 1. [file elife-90679-fig6-data1.zip › Figure 6 A-D, P Markup/Source File Figure 6A.tif]

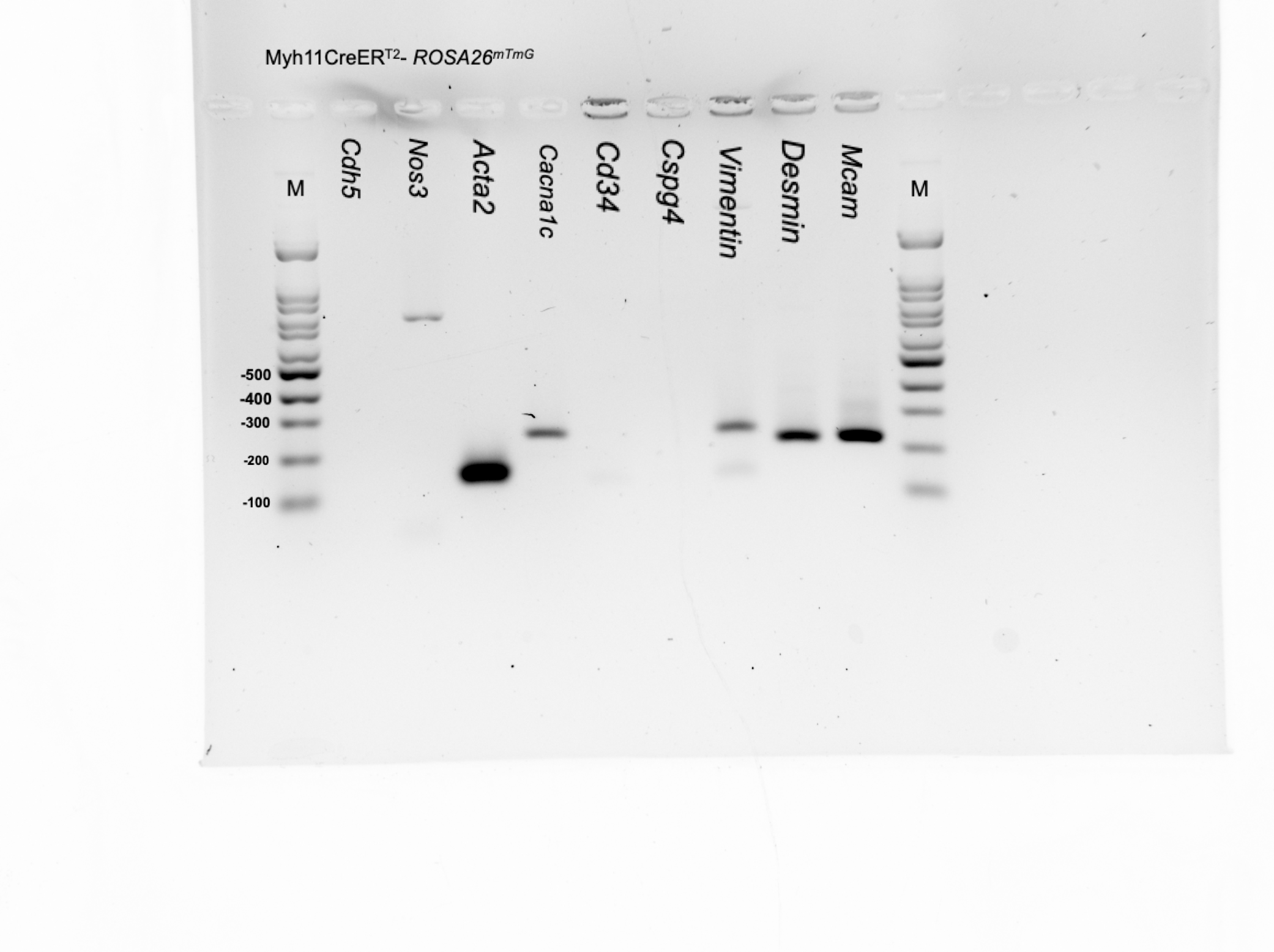

Supplement: Figure 6—source data 1. [file elife-90679-fig6-data1.zip › Figure 6 A-D, P Markup/Source File Figure 6B.tif]

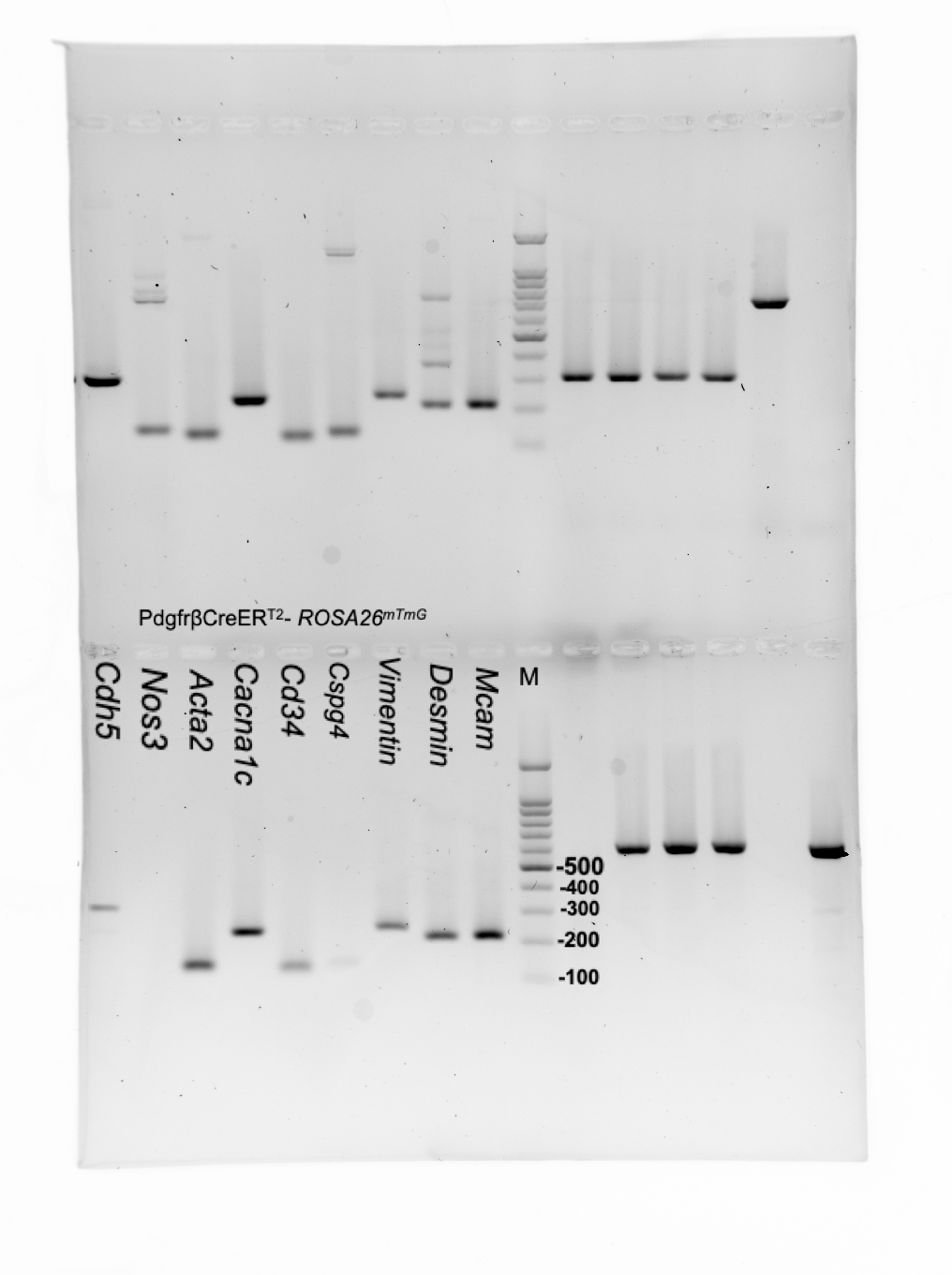

Supplement: Figure 6—source data 1. [file elife-90679-fig6-data1.zip › Figure 6 A-D, P Markup/Source File Figure 6C.tif]

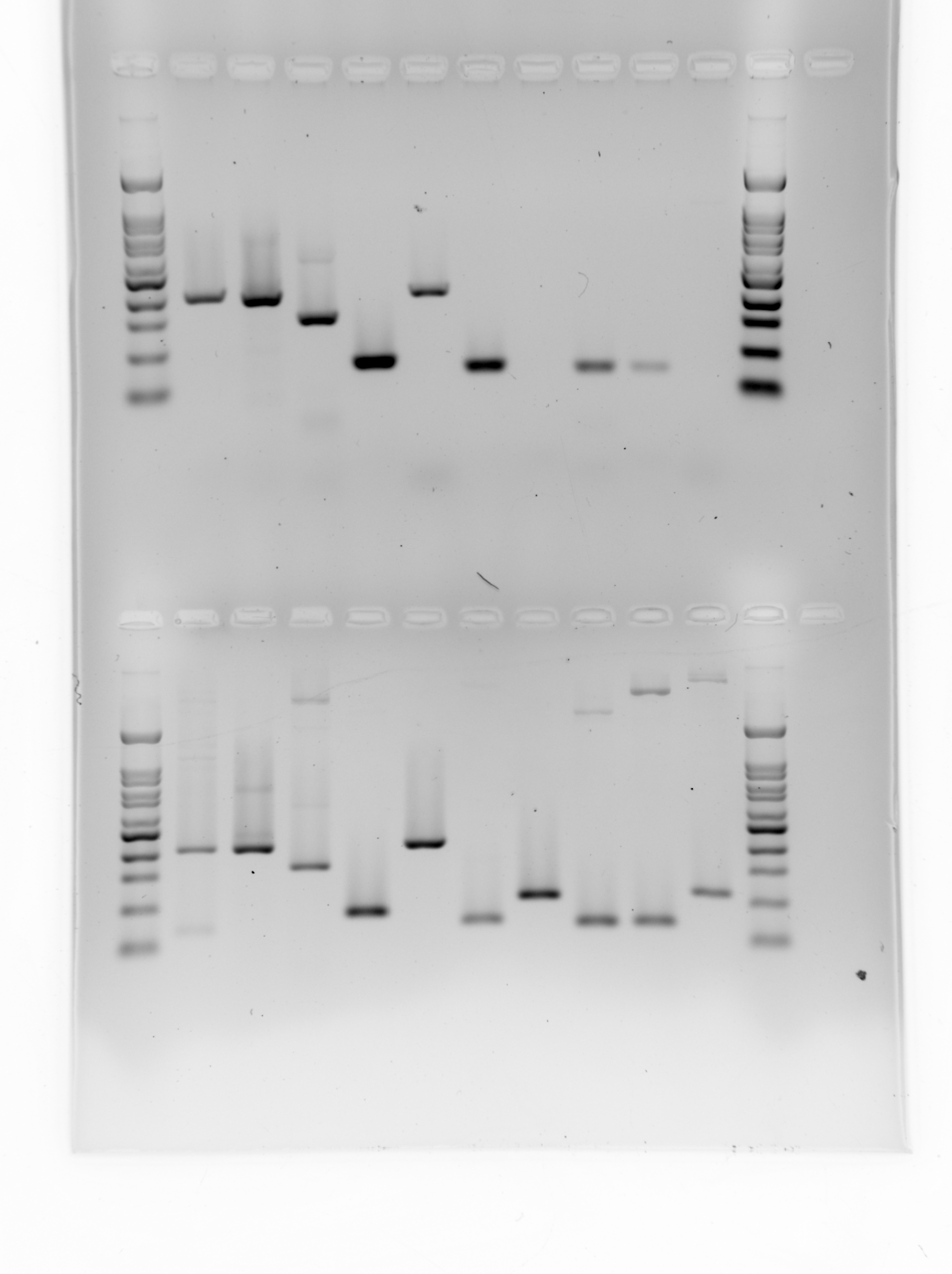

Supplement: Figure 6—source data 2. [file elife-90679-fig6-data2.zip › Figure 6 A-D, P No Markup/Source File Figure 6P.tif]

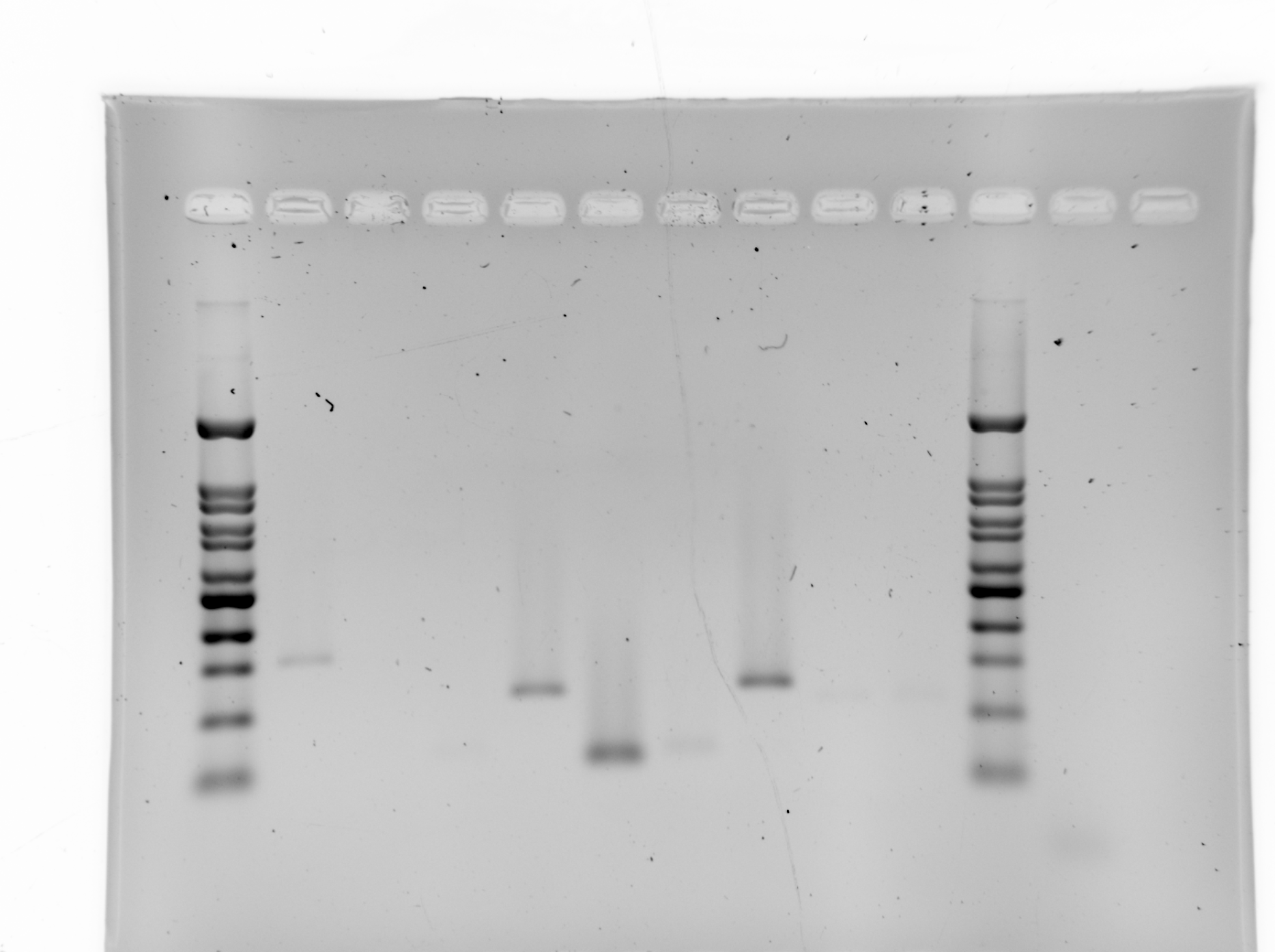

Supplement: Figure 6—source data 2. [file elife-90679-fig6-data2.zip › Figure 6 A-D, P No Markup/Source File Figure 6D.tif]

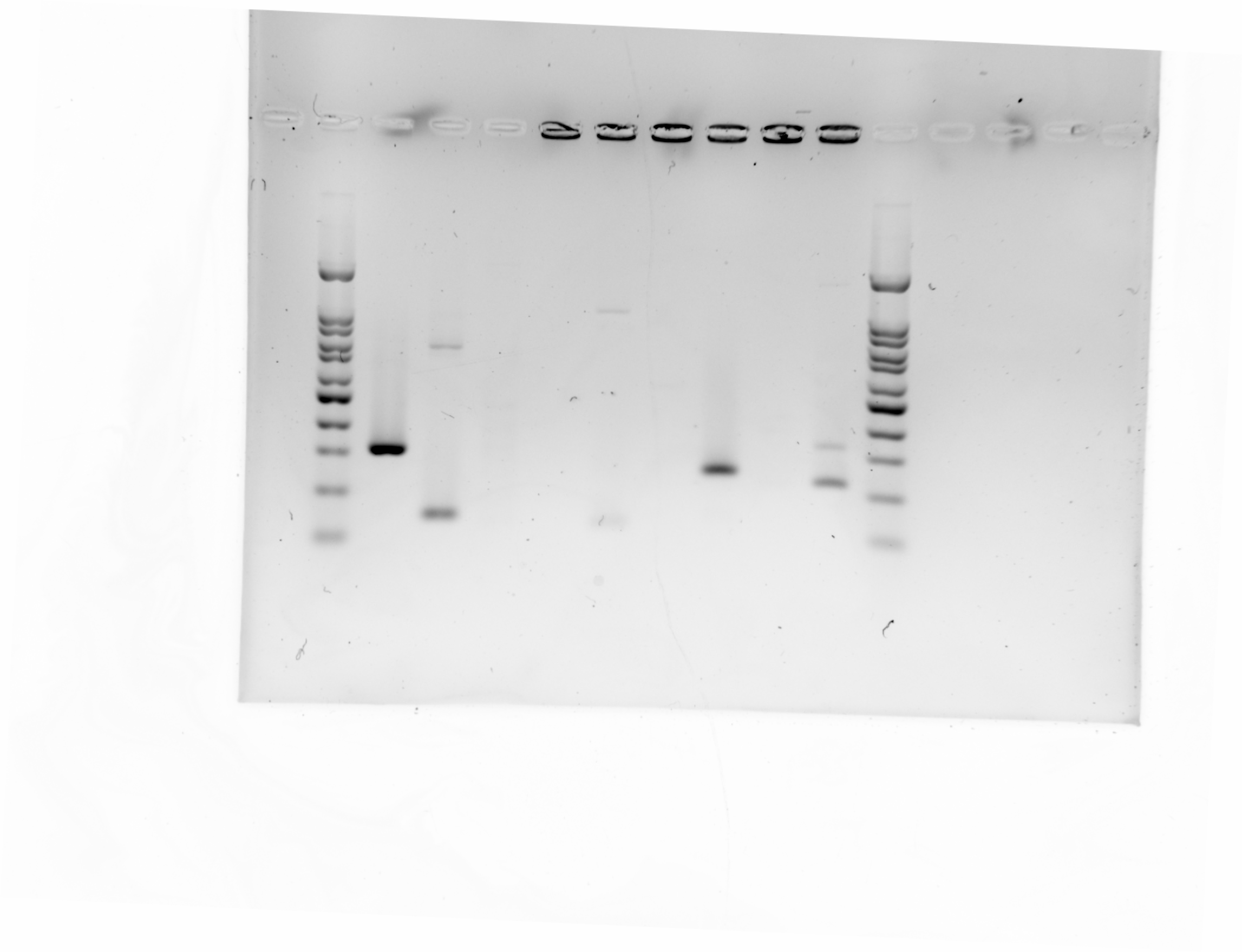

Supplement: Figure 6—source data 2. [file elife-90679-fig6-data2.zip › Figure 6 A-D, P No Markup/Source File Figure 6A.tif]

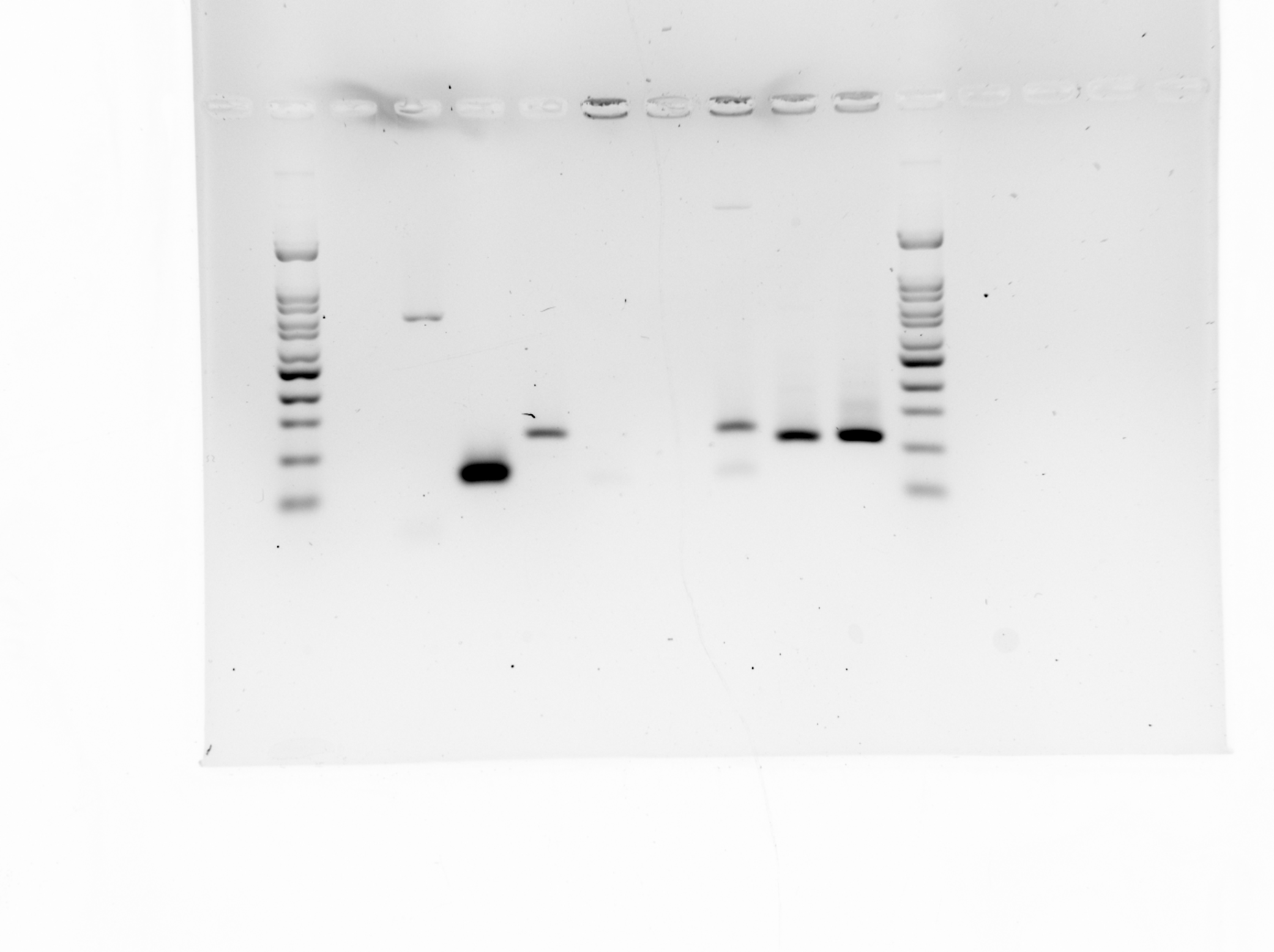

Supplement: Figure 6—source data 2. [file elife-90679-fig6-data2.zip › Figure 6 A-D, P No Markup/Source File Figure 6B.tif]

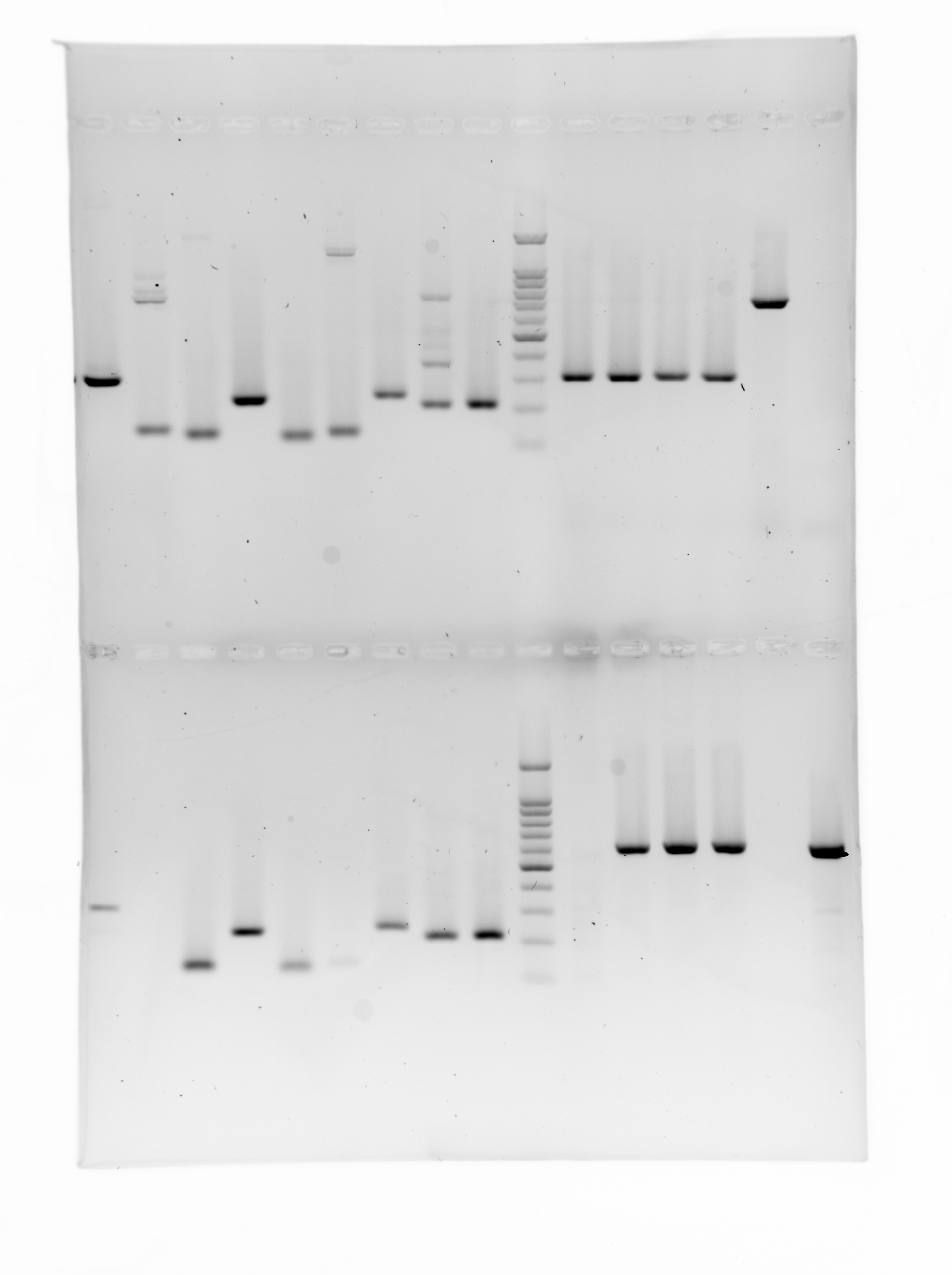

Supplement: Figure 6—source data 2. [file elife-90679-fig6-data2.zip › Figure 6 A-D, P No Markup/Source File Figure 6C.tif]

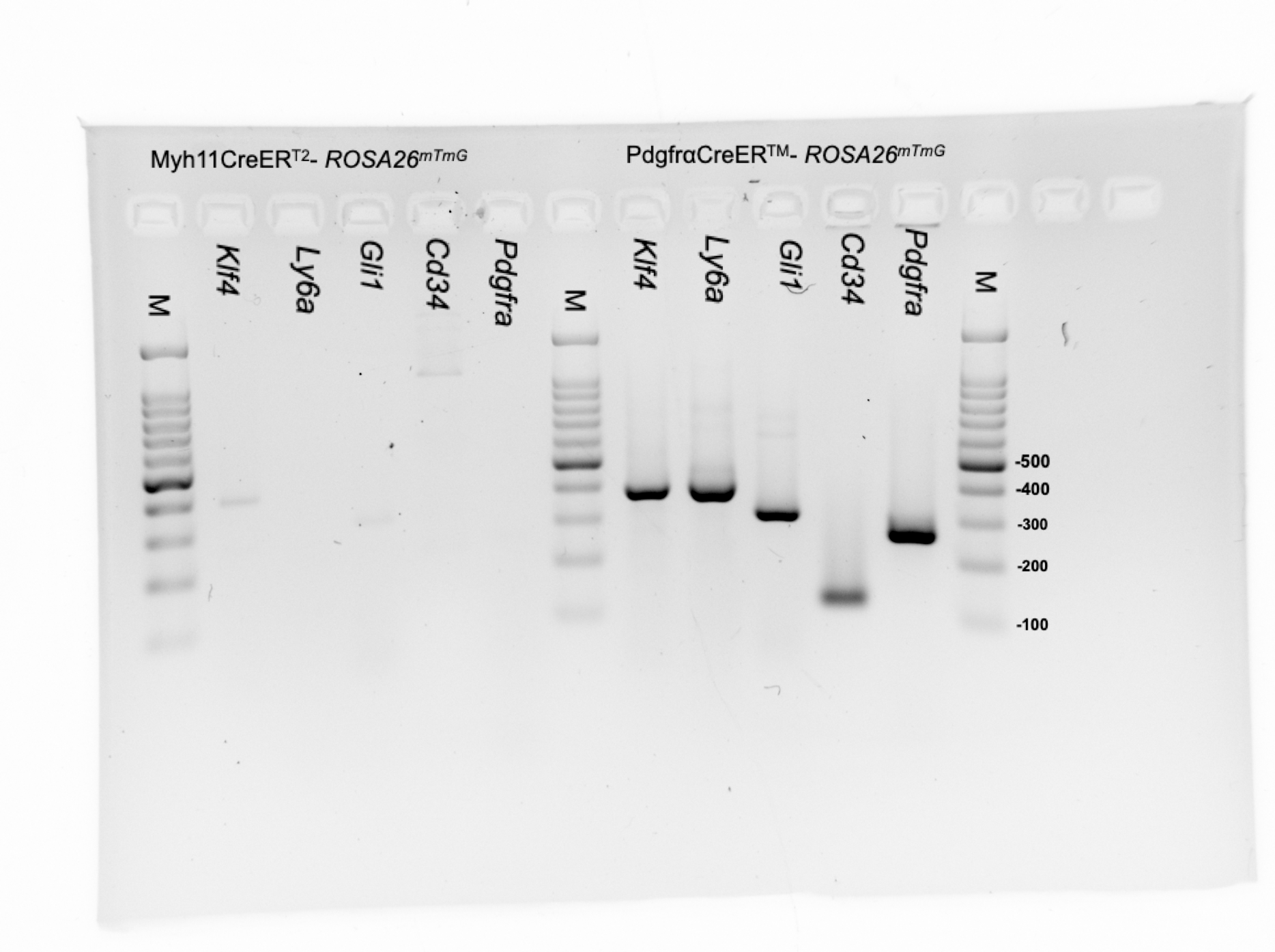

Supplement: Figure 6—figure supplement 1—source data 1. [file elife-90679-fig6-figsupp1-data1.zip › Figure 6 Supp Figure 1 A-D Markup/Source File Figure 6 Supp Figure 1A.tif]

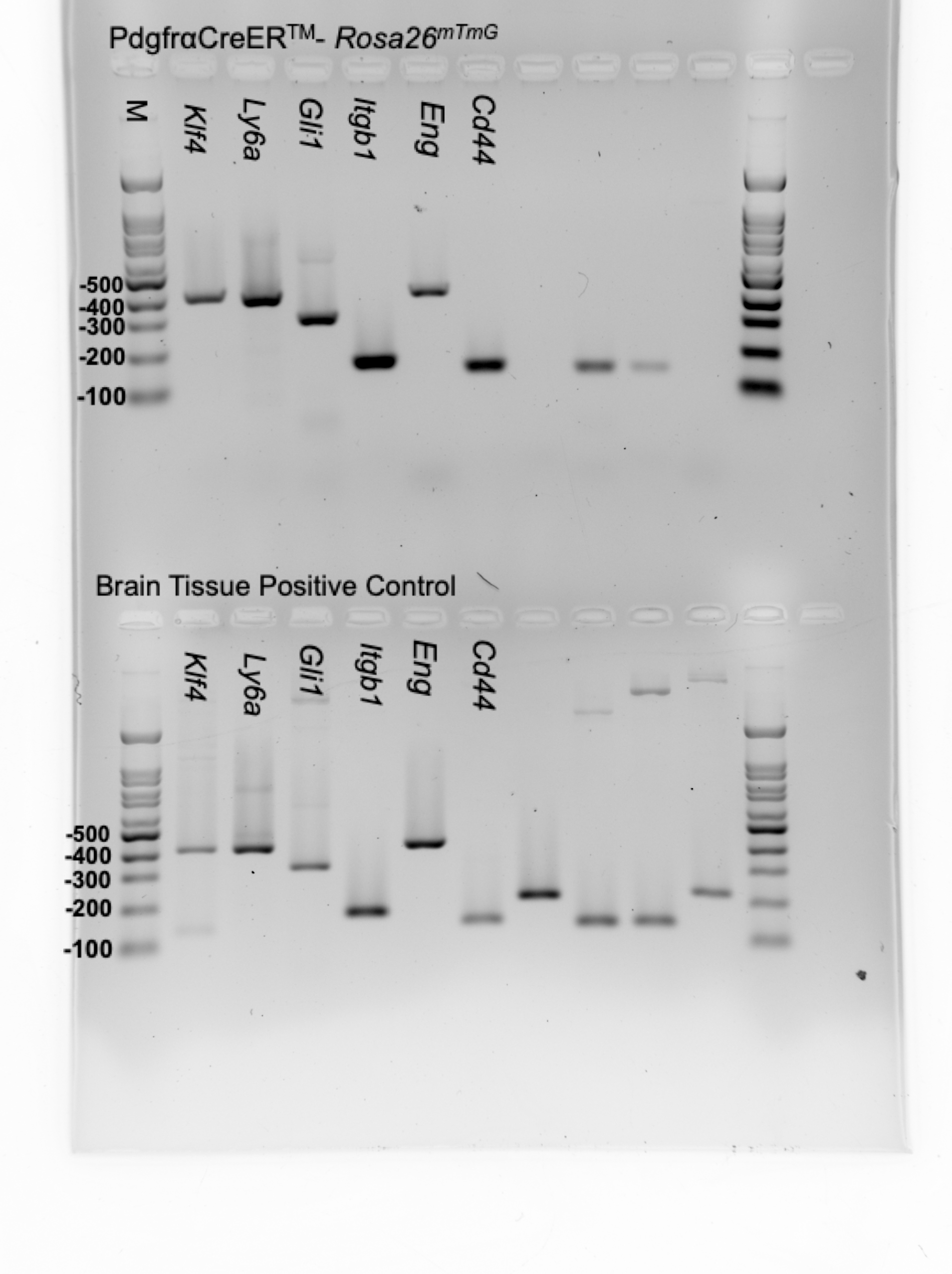

Supplement: Figure 6—figure supplement 1—source data 1. [file elife-90679-fig6-figsupp1-data1.zip › Figure 6 Supp Figure 1 A-D Markup/Source File Figure 6 Supp Figure 1C,D .tif]

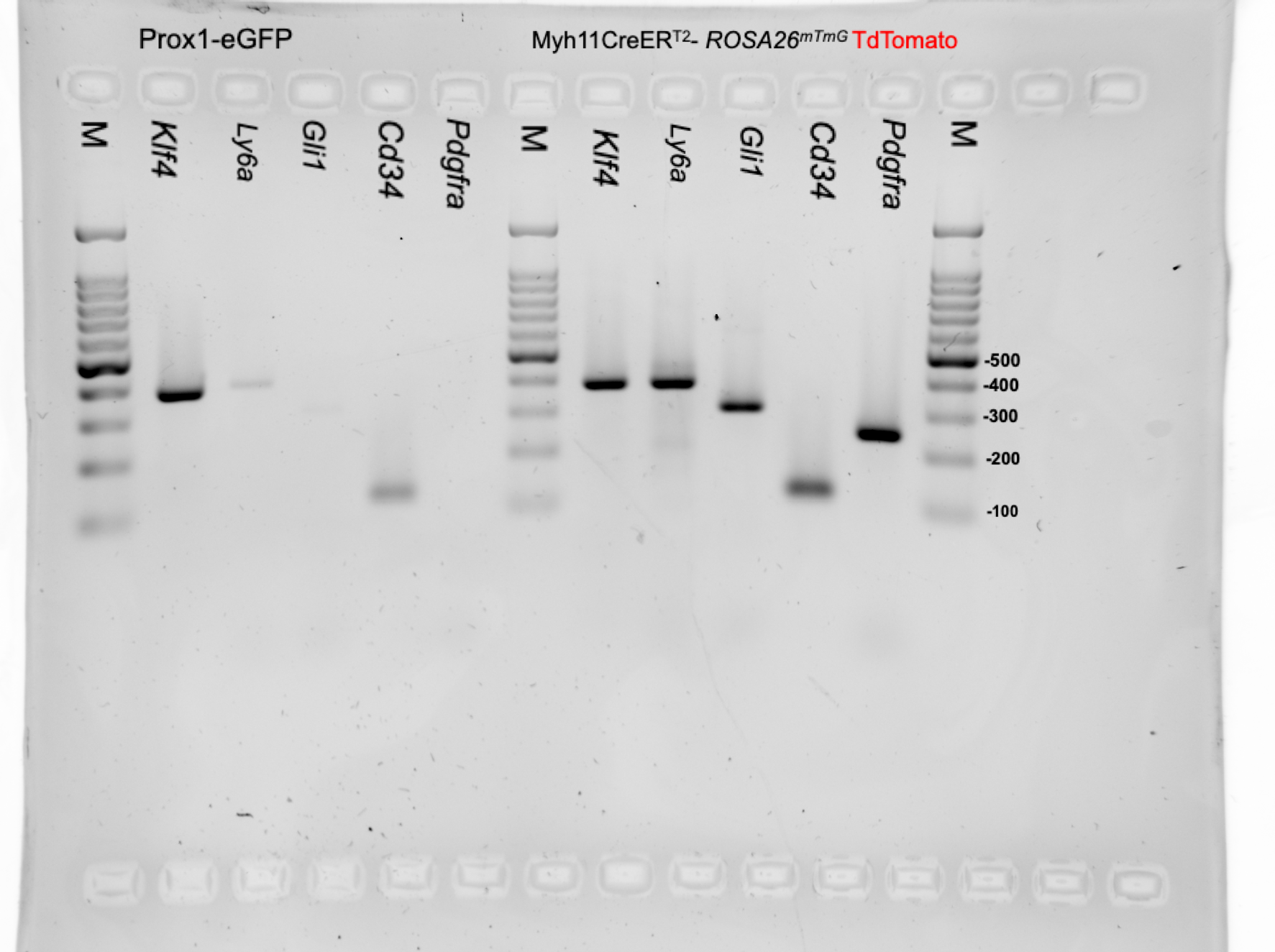

Supplement: Figure 6—figure supplement 1—source data 1. [file elife-90679-fig6-figsupp1-data1.zip › Figure 6 Supp Figure 1 A-D Markup/Source File Figure 6 Supp Figure 1B.tif]

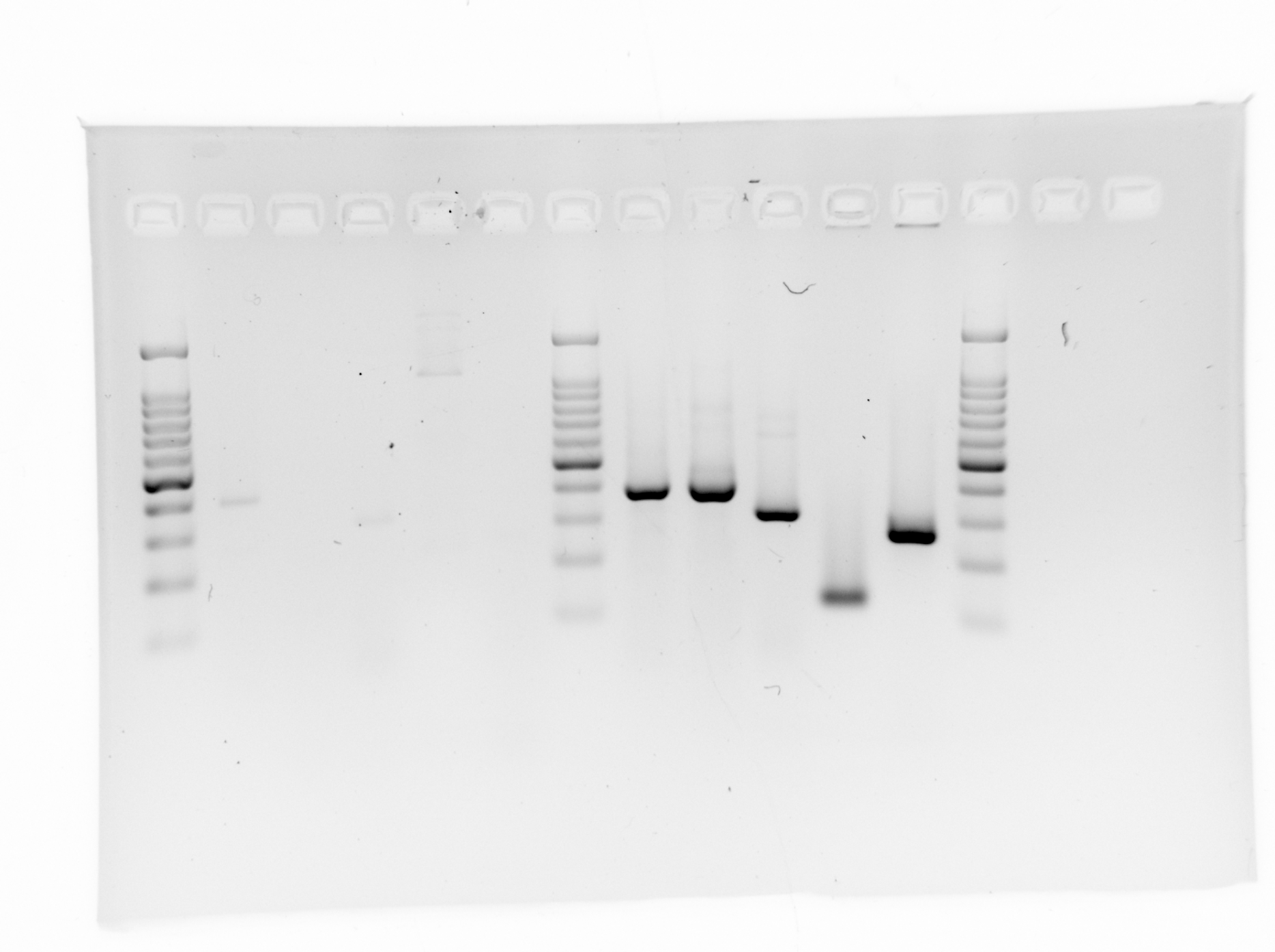

Supplement: Figure 6—figure supplement 1—source data 2. [file elife-90679-fig6-figsupp1-data2.zip › Figure 6 Supp Figure 1 A-D No Markup/Source File Figure 6 Supp Figure 1A.tif]

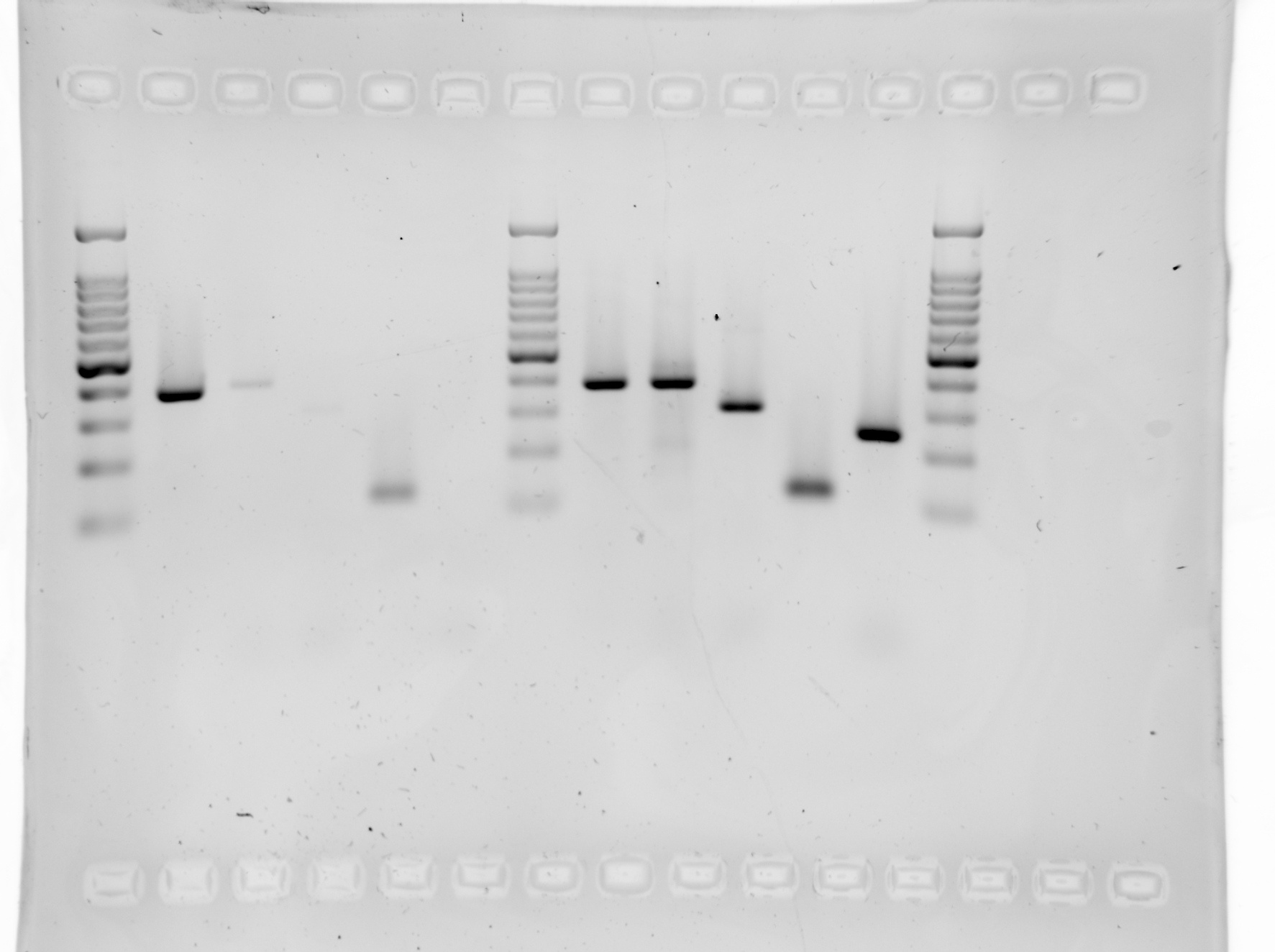

Supplement: Figure 6—figure supplement 1—source data 2. [file elife-90679-fig6-figsupp1-data2.zip › Figure 6 Supp Figure 1 A-D No Markup/Source File Figure 6 Supp Figure 1B.tif]
