# Supplementary material for: Cellular characterization of the mouse collecting lymphatic vessels reveals that lymphatic muscle cells are the innate pacemaker cells
Source: eLife. 2025 Sep 11;12:RP90679. doi: 10.7554/eLife.90679 (PMC12425481; doi:10.7554/eLife.90679)
Supplement: MDAR checklist [file elife-90679-mdarchecklist1.docx]

**Materials Design Analysis Reporting (MDAR)**

**Checklist for Authors**

**Materials:**

| **Newly created materials** | **Indicate where provided: section/figure legend** | **N/A** |
| --- | --- | --- |
| The manuscript includes a dedicated "materials availability statement" providing transparent disclosure about availability of newly created materials including details on how materials can be accessed and describing any restrictions on access. | There is a "Data Availability" section and statement on page 21. The scRNAseq dataset is uploaded to the NIH GEO: GSE277843 |  |
|  |  |  |
| **Antibodies** | **Indicate where provided: section/figure legend** | **N/A** |
| For commercial reagents, provide supplier name, catalogue number and [RRID](https://scicrunch.org/resources), if available. | Antibody information is on Page 17 in the Methods Section subtitled "Fluorescence Confocal Imaging" and are listed in the key resources table. |  |
|  |  |  |
| **DNA and RNA sequences** | **Indicate where provided: section/figure legend** | **N/A** |
| Short novel DNA or RNA including primers, probes: Sequences should be included or deposited in a public repository. | PCR primer information can be found in Table 1 on Page 32-34 and in the Key Resources Table |  |
|  |  |  |
| **Cell materials** | **Indicate where provided: section/figure legend** | **N/A** |
| Cell lines: Provide species information, strain. Provide accession number in repository OR supplier name, catalog number, clone number, OR RRID. | N/A |  |
| Primary cultures: Provide species, strain, sex of origin, genetic modification status. | N/A |  |
|  |  |  |
| **Experimental animals** | **Indicate where provided: section/figure legend** | **N/A** |
| Laboratory animals or Model organisms: Provide species, strain, sex, age, genetic modification status. Provide accession number in repository OR supplier name, catalog number, clone number, OR RRID. | Mouse strain information, age, sex, and genetic modification can be found in the Materials and Methods Section on page 14-15 | N/A |
| Animal observed in or captured from the field: Provide species, sex, and age where possible. | N/A | N/A |
|  |  |  |
| **Plants and microbes** | **Indicate where provided: section/figure legend** | **N/A** |
| Plants: provide species and strain, ecotype and cultivar where relevant, unique accession number if available, and source (including location for collected wild specimens). | N/A | N/A |
| Microbes: provide species and strain, unique accession number if available, and source. | N/A | N/A |
|  |  |  |
| **Human research participants** | **Indicate where provided: section/figure legend) or state if these demographics were not collected** | **N/A** |
| If collected and within the bounds of privacy constraints report on age, sex, gender and ethnicity for all study participants. | N/A | N/A |

**Design:**

| **Study protocol** | **Indicate where provided: section/figure legend** | **N/A** |
| --- | --- | --- |
| If the study protocol has been pre-registered, provide DOI. For clinical trials, provide the trial registration number OR cite DOI. | N/A | N/A |
|  |  |  |
| **Laboratory protocol** | **Indicate where provided: section/figure legend** | **N/A** |
| Provide DOI OR other citation details if detailed step-by-step protocols are available. | The calcium transient analysis protocol has been published: Drumm BT, Hennig GW, Baker SA, Sanders KM. Applications of Spatio-temporal Mapping and Particle Analysis Techniques to Quantify Intracellular Ca2+ Signaling In Situ. J Vis Exp. 2019 Jan 7;(143):10.3791/58989. doi: 10.3791/58989. PMID: 30663707; PMCID: PMC6996781. |  |
|  |  |  |
| **Experimental study design (statistics details) *** | | |
| **For in vivo studies: State whether and how the following have been done** | **Indicate where provided: section/figure legend. If it could have been done, but was not, write “not done”** | **N/A** |
| Sample size determination | Sample Size determination was assessed by previous experimentation of contractile indices, calcium imaging, and membrane potential recording. | N/A |
| Randomisation | There was no randomization. | N/A |
| Blinding | There was no blinding. | N/A |
| Inclusion/exclusion criteria | Page 20: Only vessels in which a recording with a minimum of 3 stable APs was successfully recorded at 2 of the 3 experimental pressures were used for subsequent analysis. | N/A |
|  |  |  |
| **Sample definition and in-laboratory replication** | **Indicate where provided: section/figure legend** | **N/A** |
| State number of times the experiment was replicated in the laboratory. | “n” values are listed in the Figure Legends. |  |
| Define whether data describe technical or biological replicates. | In some cases multiple vessels are used per mouse and this is indicated in the Figure Legends. |  |
|  |  |  |
| **Ethics** | **Indicate where provided: section/submission form** | **N/A** |
| Studies involving human participants: State details of authority granting ethics approval (IRB or equivalent committee(s), provide reference number for approval. | N/A | N/A |
| Studies involving experimental animals: State details of authority granting ethics approval (IRB or equivalent committee(s), provide reference number for approval. | Ethics statement for animal use can be found on Page 15 in the Materials and Methods section in the Subsection “Mice”. | N/A |
| Studies involving specimen and field samples: State if relevant permits obtained, provide details of authority approving study; if none were required, explain why. | N/A | N/A |
|  |  |  |
| **Dual Use Research of Concern (DURC)** | **Indicate where provided: section/submission form** | **N/A** |
| If study is subject to dual use research of concern regulations, state the authority granting approval and reference number for the regulatory approval. | N/A | N/A |

**Analysis:**

| **Attrition** | **Indicate where provided: section/figure legend** | **N/A** |
| --- | --- | --- |
| Describe whether exclusion criteria were pre-established. Report if sample or data points were omitted from analysis. If yes, report if this was due to attrition or intentional exclusion and provide justification. | An exclusion criteria for membrane potential recordings was based on having successful AP recording at two different tested pressures as stated on page 20 in the Methods subsection “Membrane Potential Recordings in IALVs“. | N/A |
|  |  |  |
| **Statistics** | **Indicate where provided: section/figure legend** | **N/A** |
| Describe statistical tests used and justify choice of tests. | The statistic test provided in the Statistics subsection of the Methods section on page 21. Statistics for scRNAseq analysis is provided on page 18. |  |
|  |  |  |
| **Data availability** | **Indicate where provided: section/submission form** | **N/A** |
| For newly created and reused datasets, the manuscript includes a data availability statement that provides details for access (or notes restrictions on access). | The data availability statement is provided on page 21.The data availability for the generated scRNAseq dataset is found on page 21. NCBI GEO: GSE277843. |  |
| When newly created datasets are publicly available, provide accession number in repository OR DOI and licensing details where available. | The accession number for NCBI GEO is provided on page 21. | N/A |
| If reused data is publicly available provide accession number in repository OR DOI, OR URL, OR citation. | N/A | N/A |
|  |  |  |
| **Code availability** | **Indicate where provided: section/figure legend** | **N/A** |
| For any computer code/software/mathematical algorithms essential for replicating the main findings of the study, whether newly generated or re-used, the manuscript includes a data availability statement that provides details for access or notes restrictions. | N/A | N/A |
| Where newly generated code is publicly available, provide accession number in repository, OR DOI OR URL and licensing details where available. State any restrictions on code availability or accessibility. | N/A | N/A |
| If reused code is publicly available provide accession number in repository OR DOI OR URL, OR citation. | N/A | N/A |

**Reporting:**

The MDAR framework recommends adoption of discipline-specific guidelines, established and endorsed through community initiatives.

| **Adherence to community standards** | **Indicate where provided: section/figure legend** | **N/A** |
| --- | --- | --- |
| State if relevant guidelines (e.g., ICMJE, MIBBI, ARRIVE, STRANGE) have been followed, and whether a checklist (e.g., CONSORT, PRISMA, ARRIVE) is provided with the manuscript. | N/A | N/A |
